# Redox State of Epigallocatechin Gallate Modulates Its Inhibition of Acrylamide Formation via the 3-Aminopropionamide Pathway

**DOI:** 10.3390/foods15101781

**Published:** 2026-05-18

**Authors:** Yajing Qi, Mengjie Gao, Jiahao Cheng, Tianxiang Yang, En Han, Bin Xu

**Affiliations:** School of Food and Biological Engineering, Jiangsu University, Zhenjiang 212013, China; xiaoyataoyao@163.com (Y.Q.); 13905521905@163.com (M.G.); chengjh2085@163.com (J.C.); 13130959645@163.com (T.Y.); en_han@126.com (E.H.)

**Keywords:** epigallocatechin gallate, acrylamide, 3-aminopropionamide, Maillard reaction

## Abstract

This study aimed to elucidate the impact of environmental factors on the efficacy of epigallocatechin gallate (EGCG) in inhibiting acrylamide formation and to clarify the role of the 3-aminopropionamide (3-APA) pathway in this process. Asparagine–glucose and 3-APA model systems were employed for the investigation. The results revealed that EGCG exerted a pronounced, condition-dependent inhibitory effect on acrylamide formation during the Maillard reaction. The maximum inhibition rate of 91% was observed at 180 °C and pH 6.0 without metal ions, while alkaline conditions, excessive heating, and Fe^3+^ markedly weakened the inhibitory capacity of EGCG. In the 3-APA model, a positive correlation (R^2^ = 0.9111) was found between acrylamide generation and EGCG oxidation, and the key *o*-quinone-derived adduct was identified as an indirect evidence for EGCG oxidation. Collectively, the redox state of EGCG, which is highly susceptible to food processing conditions, may modulate its anti-acrylamide activity. These findings provide valuable mechanistic insights for the rational application of EGCG to mitigate acrylamide contamination in thermally processed foods.

## 1. Introduction

Acrylamide exhibits neurotoxicity and genotoxicity and may potentially induce cancer. Based on animal experimental data, the International Agency for Research on Cancer (IARC) classified acrylamide as a Group 2A probable human carcinogen in 1994 [1,2]. The Joint Expert Committee on Food Additives (JECFA) identified potato chips, coffee, pastries, and sweet biscuits as major dietary sources of acrylamide worldwide, particularly in carbohydrate-rich foods cooked at temperatures exceeding 120 °C [3]. The formation of acrylamide during food processing is a complex multi-factor process, primarily involving the Maillard reaction and lipid oxidative degradation. The Maillard reaction not only generates acrylamide but also contributes to the desirable flavors and colors of many food products, such as bread, coffee, and chocolate, through the production of volatile aroma compounds and pigment molecules [4,5,6]. Among the various pathways, the reaction between the amino group of asparagine (Asn) and the carbonyl group of reducing compounds constitutes the major route, known as the asparagine pathway. This reaction process consists of several steps. First, a carbonyl compound reacts with Asn to form a Schiff base. Then, decarboxylation takes place to produce an azomethine ylide. This intermediate can either directly convert into acrylamide or form acrylamide through the deamination of 3-aminopropionamide (3-APA). It is noteworthy that 3-APA serves as a critical precursor for acrylamide formation. Under suitable temperature and pH conditions, its deamination reaction can contribute up to 60% of the molar yield of acrylamide [7,8]. Several factors influence acrylamide formation in food, including the contents of free asparagine and reducing sugars, food processing parameters like temperature and time, and the pH and water activity of the food systems [9].

Adding polyphenol inhibitors is an effective way to inhibit acrylamide formation in foods. However, existing research presents inconsistent findings regarding the inhibitory effect of EGCG, with some studies reporting neutral or even promotive effects [10,11]. For instance, Huang et al. [10] observed that in a low-moisture model system simulating potato processing (asparagine-glucose-potato powder), the addition of EGCG inhibited acrylamide formation by 78.2% at the optimal concentration. However, when the concentration exceeded 500 μmol/L, it significantly promoted acrylamide formation. Similarly, in yeast doughnuts fortified with EGCG-rich green tea extract, the acrylamide content decreased by 15% and 10% at addition levels of 0.25% and 0.5%, respectively. In contrast, when the addition level reached 1%, the acrylamide content increased significantly by 120% [11]. These results indicate that the inhibitory effect of EGCG on acrylamide formation may be influenced by multiple factors, such as concentration, processing conditions, and system composition. Nevertheless, the underlying mechanisms and strategies to mitigate the conditional promotive effect of EGCG on acrylamide generation remain unclear and require further investigation.

The primary mechanism by which EGCG inhibits acrylamide formation is its well-established capacity to scavenge reactive carbonyl compounds produced during the Maillard reaction [12]. Specifically, EGCG suppresses acrylamide generation by trapping reactive carbonyl species such as methylglyoxal and glyoxal, thereby blocking their reaction pathway with asparagine. Studies have confirmed that this trapping occurs through electrophilic substitution reactions on the A ring of the EGCG molecule, where the C-6 and C-8 sites are attacked by carbonyl compounds [13,14,15]. While these inhibitory pathways are documented, recent evidence suggests a more complex dual role where flavan-3-ols like epicatechin (EC) can oxidize to quinones and subsequently promote acrylamide formation via the 3-APA pathway [16]. However, current knowledge remains largely confirmatory regarding the existence of this promotive effect and fails to explain why EGCG displays such inconsistent performance across different food matrices. There is a critical knowledge gap concerning how specific environmental triggers such as temperature, pH, and metal ions dictate the role of EGCG in the 3-APA pathway. This uncertainty hinders the practical application of flavan-3-ols and prevents the development of precision mitigation strategies in food processing.

Therefore, we propose the scientific hypothesis that environmental factors impact the acrylamide-inhibitory efficacy of EGCG partially by modulating its oxidation to quinone and the subsequent reaction with 3-APA. This work systematically investigates how temperature, pH, and metal ions affect the 3-APA-mediated pathway by combining model system kinetics with electrochemical and chromatographic and mass spectrometric techniques. The findings are expected to elucidate the underlying mechanism and provide strategies for optimizing the application of EGCG to mitigate acrylamide formation.

## 2. Materials and Methods

### 2.1. Chemicals

Acrylamide, epigallocatechin gallate (EGCG), and β-alaninamide hydrochloride were purchased from Shanghai Yuanye Bio-Technology Co., Ltd. (Shanghai, China). Glucose, asparagine, disodium hydrogen phosphate, potassium dihydrogen phosphate, ferric chloride, copper chloride, and magnesium chloride were obtained from Sinopharm Chemical Reagent Co., Ltd. (Shanghai, China). o-Phenylenediamine (OPD) was acquired from Shanghai Aladdin Biochemical Technology Co., Ltd. (Shanghai, China). Methanol and acetonitrile were supplied by Tedia Company (Zhenjiang, China).

### 2.2. Preparation of Asparagine-Glucose Maillard Reaction System

The method was adapted from that described by Xu et al. [17] with minor modifications. Solutions containing 20 mmol/L glucose, 20 mmol/L asparagine, and 5 mg/mL EGCG were prepared with 0.1 M phosphate buffer (pH 7.0). For each reaction, 100 µL of glucose, 100 µL of asparagine, and 50 µL of EGCG solution (or pure water as control) were combined in an open glass tube and mixed. The tube was then immersed in an oil bath preheated to 180 °C. It was removed after 5 min of reaction and immediately cooled. After cooling, 1 mL of ultrapure water was added, and the mixtures were ultrasonicated for 10 min. The extract was filtered through a 0.22 µm membrane, and the resulting filtrate was subjected to acrylamide quantification.

The impact of reaction temperature, pH, and metal ions on the acrylamide formation was evaluated individually. For reactions at different temperatures, the pH of the phosphate buffer was set at 7.0 and no metal ion was added, with heating at 180, 200, and 220 °C. For reactions at different pH, the phosphate buffers were prepared at pH 6.0, 7.0, and 8.0 without other metal ions, and the samples were heated at 180 °C. The pH values were those of the buffer before heating. As for the reactions with different metal ions, 0.001 M Fe^3+^, Cu^2+^, and Mg^2+^ were incorporated in the system at a fixed pH of 7.0 and a temperature of 180 °C.

### 2.3. Preparation of the Reaction System of 3-APA and EGCG

Solutions of 1 mmol/L 3-APA and EGCG at concentrations of 0, 0.5, and 5 mg/mL were prepared in 0.1 M phosphate buffer (pH 7.0). For each test tube, 200 µL of 3-APA solution and 50 µL of EGCG solution were added sequentially. The open tubes were then heated in an oil bath at 180 °C and removed after 1, 2, 3, 4, 5, 7, or 10 min of reaction, followed by immediate cooling. After cooling, 1 mL of ultrapure water was added to each tube, and ultrasonic extraction was carried out for 10 min. The extracts were filtered through a 0.22 μm membrane, and the filtrates were used for quantitative analysis of acrylamide and 3-APA. For tubes reacted for 10 min and cooled, 0.2 mL of methanol was added and the mixture was vortex-mixed to dissolve, then filtered through a 0.22 μm membrane. The resulting filtrate was used for adduct identification. Detailed procedures for investigating the effects of temperature, pH, and metal ions on the reaction kinetics were the same as those in Section 2.2.

### 2.4. Analysis of Acrylamide

The analysis was performed using a LC-20AT chromatograph equipped with an SPD-20A detector (Shimadzu, Shanghai, China). Separation was achieved on an XSelect HSS T3 column (4.6 mm × 250 mm, 5 µm) maintained at 35 °C. The mobile phase consisted of water (Phase A) and acetonitrile (Phase B) with a flow rate of 1 mL/min. The gradient elution program was set as follows. The initial condition was 0% B from 0 to 8 min. The proportion of B was then increased to 99% from 8 to 10 min and held until 15 min. Subsequently, the gradient returned to 0% B from 15 to 17 min and was maintained until 23 min for column re-equilibration. The detection wavelength was set at 205 nm [16].

No interfering peak was observed at the characteristic retention time of acrylamide. The resolution between acrylamide and adjacent impurity peaks was 2.09, with LOD and LOQ calculated as 17.6 ng/mL and 58.6 ng/mL, respectively. These validation parameters confirm the specificity and reliability of the current chromatographic method for accurate acrylamide quantification in this specific model system.

### 2.5. Analysis of Redox State of EGCG

The analytical method was adapted from previous literature with minor modifications [18]. A traditional three-electrode system (CHI 660D electrochemical workstation, Shanghai Chen Hua Instrument Co., Ltd., Shanghai, China) was used. A glassy carbon electrode served as the working electrode, alongside a saturated calomel reference electrode and a platinum mesh counter electrode. Prior to each individual measurement, the glassy carbon electrode was polished with alumina powder, rinsed thoroughly with ultrapure water, and dried under a stream of nitrogen gas to prevent surface contamination. The platinum mesh electrode was cleaned via ultrasonic treatment in ultrapure water for 5 min, and the saturated calomel electrode was equilibrated to ensure stability.

The cooled reaction mixtures in Section 2.3 were uniformly diluted to 3 mL before filtration through a 0.22 µm microporous membrane and then used for DPV measurement. The operating parameters were set as follows: pulse amplitude = 50 mV, pulse width = 0.2 s, step potential = 5 mV, scan rate = 10 mV/s, a potential range from −0.20 to 0.50 V, and a residence time of 2 s. The repeatability of peak area measurements was verified by parallel experiments (n = 3), and the relative standard deviation (RSD) was ≤2.6%, indicating good repeatability of the DPV method.

### 2.6. Analysis of EGCG-Quinone-OPD

Trapping of EGCG quinone with OPD was performed according to a previously reported method by Tan et al. [19] with slight modifications. Briefly, the sample obtained from Section 2.3 was treated with 40 μL of a 10 mg/mL OPD in methanol solution and allowed to react for 30 min. The resulting mixture was filtered through a 0.22 μm membrane prior to mass spectrometric analysis.

Liquid chromatography-mass spectrometry (LC-MS) analysis was carried out on a Thermo Fisher Q Exactive plus system equipped with a HYPERSIL GOLD Vanquish analytical column (2.1 mm ×100 mm, 1.9 μm). The column temperature was maintained at 30 °C. The mobile phase consisted of water (Phase A) and methanol (Phase B), delivered at a flow rate of 0.3 mL/min. The gradient elution program was set as follows: 0–15 min, 5–25% B; 15–20 min, 25–40% B; 20–22 min, 40–80% B; 22–24 min, 80–100% B; 24–27 min, 100% B; 27–30 min, 100–5% B.

Mass spectrometry was performed in negative ion mode with electrospray ionization (ESI). The capillary voltage was set to 3.2 kV and the cone voltage was 30 V. The ion source temperature was maintained at 100 °C and the capillary temperature at 320 °C. For MS/MS analysis, the cone voltage remained at 30 V, and the collision energy was set to 20 and 30 eV. All acquired mass spectrometry data were processed using Xcalibur 4.2 software.

### 2.7. Kinetic Analysis

The consumption of EGCG and the formation of acrylamide in the samples from Section 2.3 were quantified. The accumulation phase of acrylamide content was analyzed using a zero-order kinetic model (Equation (1)), while the consumption process of EGCG was modeled with a first-order kinetic equation (Equation (2)). Based on these models, the reaction rate constant k under each processing condition was determined.

(1) $$C_{acrylamide}=C_{acrylamide0}+k_{acrylamide}t$$

(2) $$lnC_{EGCG}=lnC_{EGCG0}{-k}_{EGCG}t$$

where *C*_acrylamide_ and *C*_EGCG_ are the concentrations of acrylamide and EGCG at time *t*, while *C*_acrylamide0_ and *C*_EGCG0_ are their initial concentrations. The parameters *k*_acrylamide_ and *k*_EGCG_ represent the apparent rate constants for acrylamide formation and EGCG consumption, respectively, and t is the heating time.

### 2.8. Statistical Analysis

All experiments were conducted independently in triplicates with a sample size of n = 3 for each treatment group, and the obtained data were expressed as the mean and standard deviation. Data processing and statistical analyses were executed using Excel 2016 and SPSS 26, whereas Origin 2022 was utilized for plotting. Prior to conducting the analysis of variance, the fundamental assumptions of data normality and homogeneity of variance were verified. Exact *p* values alongside effect sizes (η_p_^2^) were reported to evaluate both the statistical significance and the relative contribution for each experimental factor. A probability level of *p* < 0.05 was defined as statistically significant, and Duncan’s multiple range test was applied to compare the differences among group means.

## 3. Results and Discussion

### 3.1. Effect of Conditions on the Inhibition of Acrylamide Formation by EGCG in the Maillard Reaction

**Figure 1:**
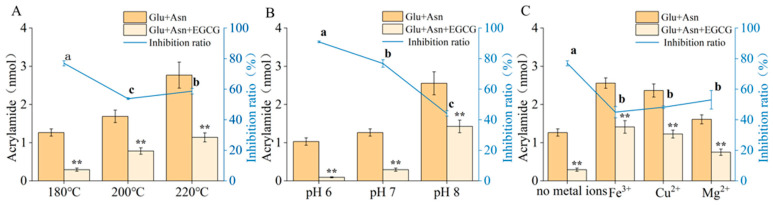
Effect of EGCG on the formation of acrylamide and the inhibition ratio in the asparagine–glucose Maillard reaction model system under different conditions. ** denotes a significant difference (*p* < 0.05) in acrylamide formation between the Glu + Asn group and the Glu + Asn + EGCG group. Different lowercase letters indicate significant differences (*p* < 0.05) in inhibition rates among different conditions. Effect of EGCG on the formation of acrylamide and the inhibition ratio in the asparagine–glucose Maillard reaction model system under different temperatures (**A**), pH conditions (**B**), and with different kinds of metal ions (**C**).

An asparagine–glucose model system was employed to simulate the formation of acrylamide in the Maillard reaction during food processing. EGCG was introduced into this system, and the mixture was heated for 5 min under various reaction conditions. Acrylamide formation was quantified, and the results are presented in Figure 1.

Reaction conditions exerted a significant influence on both the formation of acrylamide in the Maillard reaction and the inhibitory efficacy of EGCG. High temperature, elevated pH, and the presence of metal ions all promoted acrylamide formation. While EGCG consistently inhibited acrylamide formation across all conditions, the extent of inhibition varied with the environment. In systems with temperatures of 180–220 °C, EGCG displayed the strongest inhibitory effect at 180 °C, achieving an inhibition rate of approximately 77%. As temperature increased, the inhibitory effect diminished, with the inhibition rate declining to between 54% and 59%. In systems buffered at different pH levels, the inhibitory activity of EGCG followed the order of pH 6 > pH 7 > pH 8. The highest inhibition rate, reaching 91%, was observed at pH 6, whereas the weakest inhibition, only 44%, occurred at pH 8. Compared to the control group without metal ions, the addition of Fe^3+^, Cu^2+^, or Mg^2+^ all reduced the inhibitory effect of EGCG on acrylamide formation.

The promoting effect of high temperature and high pH on acrylamide formation in Maillard reactions has been extensively documented. Practical strategies such as controlling heating intensity and incorporating organic acids have been employed to mitigate acrylamide accumulation [3,20,21]. The impact of metal ions on acrylamide formation in the Maillard reaction is complex, influenced by ion species and the type of carbonyl compounds, and may result in either inhibition or promotion [22]. In contrast, the effect of reaction conditions on the acrylamide-inhibiting activity of polyphenols like EGCG has received limited research attention. This study demonstrates that the inhibitory efficiency of EGCG is highly sensitive to environmental conditions. We hypothesize that this phenomenon may be partially associated with condition-dependent changes in EGCG stability and redox characteristics. Such variations may further interfere with its carbonyl-scavenging capacity in the Maillard system and its oxidative transformation in the 3-APA pathway. The potential correlation between EGCG redox behavior and 3-APA metabolism is further explored in the subsequent section.

### 3.2. Effect of Conditions on the Conversion of 3-APA to Acrylamide in the Presence of EGCG

#### 3.2.1. Effect of Temperature on the Conversion of 3-APA to Acrylamide in the Presence of EGCG

**Figure 2:**
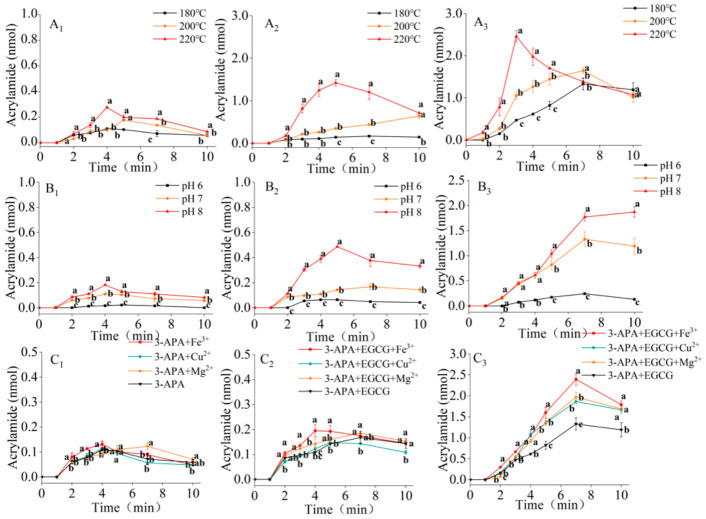
The amount of acrylamide produced in the reaction of 3-APA and EGCG at different temperatures (**A_1_**–**A_3_**), pH conditions (**B_1_**–**B_3_**), and with different kinds of metal ions (**C_1_**–**C_3_**). The subscript 1, 2, and 3 denote the control group (3-APA only), the EGCG low addition group with a molar ratio of 3-APA:EGCG at 1:0.43, and the EGCG high addition group with a molar ratio of 3-APA:EGCG at 1:4.3, respectively. Different letters denote significant differences at the same time points (*p* < 0.05).

Figure 2A shows that the influence of EGCG on acrylamide formation exhibited notable dependence on both temperature and addition dosage. At the lower temperature (180 °C) and with a low dose of EGCG, the promoting effect was relatively mild. In the system with low-dose EGCG, the acrylamide content continued to increase over 10 min at 180 °C and 200 °C, while at 220 °C, it exhibited an initial increase followed by a decrease. The maximum acrylamide yields under these conditions were 1.5, 4.1, and 5.2 times higher than that of the control group (3-APA heated alone), respectively. In the system supplemented with a high dose of EGCG, the acrylamide level initially increased and then decreased within 10 min at all three temperatures (180 °C, 200 °C, and 220 °C). The maximum acrylamide amounts reached 1.40, 1.65, and 2.46 nmol, respectively, which corresponded to 12.7, 10.3, and 9.5 times the maximum yield observed in the control group.

This declining trend at the later heating stage was closely related to the intrinsic instability of newly generated acrylamide. Acrylamide produced during thermal processing is thermally labile and prone to continuous degradation under sustained high-temperature environments. Excessive heating facilitates its deamination and molecular chain cleavage, while reactive carbonyl substances and phenolic metabolites in the reaction system further consume acrylamide through nucleophilic addition and oxidative reactions, collectively resulting in the loss of formed acrylamide [23,24,25,26]. Therefore, the final acrylamide accumulation is likely modulated by the dynamic equilibrium between continuous generation and thermally induced degradation during heating.

Elevated temperature may accelerate the oxidation of EGCG to form *o*-quinone, which could potentially contribute to the reaction between *o*-quinone and 3-APA to generate acrylamide. This potential promoting effect can be mitigated by selecting specific mild conditions, such as lower temperatures. Temperature is a key factor that may influence both the formation and reactivity of *o*-quinones. High-temperature environments facilitate the generation of *o*-quinones and enhance their participation in subsequent reactions, a phenomenon consistent with observations from various food processing studies. For instance, increasing the processing temperature of green tea from 85 °C to 120 °C promoted the formation of catechin quinones and their subsequent polymerization, leading to significant darkening of the tea liquor [27]. Similarly, during cocoa bean drying, elevated temperatures trigger non-enzymatic oxidation of phenolic compounds, initiating molecular condensation reactions via *o*-quinone intermediates [28]. Furthermore, temperature influenced the dynamic balance of acrylamide through a dual mechanism. Under high-temperature conditions, it not only promoted the formation of acrylamide but also accelerated its degradation [24,26]. As a result, the production of acrylamide often exhibits a parabolic trend, increasing initially and then decreasing with prolonged reaction time.

#### 3.2.2. Effect of pH Value on the Conversion of 3-APA to Acrylamide in the Presence of EGCG

As shown in Figure 2B, all systems exhibited typical kinetic behavior characterized by an initial increase followed by a decrease over reaction time, except for the high-dose EGCG system at pH 8, where the acrylamide accumulation period was extended. Consistent with observations under different temperature conditions, a clear dose-dependent effect was evident across pH values. In the EGCG low addition systems, the maximum acrylamide yields were 1.5 to 3.0 times higher than that of the control group (3-APA heated alone). In the EGCG high addition systems, the maximum acrylamide production reached 0.25, 1.40, and 1.87 nmol at pH 6, 7, and 8, respectively. Compared to the 3-APA-only system, these values represented significant increases in acrylamide content by 10.4 to 12.7 times.

pH is regarded as a vital environmental factor that may modulate the conversion of 3-APA to acrylamide in the presence of EGCG, potentially through two major pathways. First, acidic pH conditions may restrict *o*-quinone production, which could further slow the interaction between *o*-quinone species and 3-APA, thereby potentially limiting subsequent acrylamide generation. The influence of pH on *o*-quinone formation was particularly pronounced in the oxidation pathways of phenolic compounds. Dong et al. [29] developed a catechin/epicatechin/chlorogenic acid oxidation model system and quantitatively demonstrated that pH exerted a stronger influence than polyphenol type or temperature, underscoring its critical role. Under acidic conditions, the non-enzymatic oxidation rate of catechin was significantly reduced, and the chemical oxidation of phenolic compounds was likewise hindered [30]. Likewise, the slower electron transfer in polyphenols under acidic conditions further supports the view that low pH can potentially constrain *o*-quinone formation [31]. Second, pH affected the speciation of 3-APA in the system, which in turn influenced acrylamide generation. At lower pH values, a greater proportion of nucleophilic unprotonated amine groups (-NH_2_) are converted into non-nucleophilic protonated amines (-NH_3_^+^). This reduced nucleophilic attack capacity inhibited the reaction, leading to acrylamide formation [32]. Studies have shown that in fried potato chip models, acid treatment could significantly suppress acrylamide formation. Soaking potatoes in a low-pH solution protonated the α-amino group (-NH_3_^+^) of asparagine, thereby inhibiting its reaction with reducing sugars and effectively blocking the initial stage of the Maillard reaction [33]. Analogously, 3-APA mainly exists in a protonated state under acidic conditions, which may reduce its reactivity with EGCG oxidation products and limit the subsequent formation of acrylamide. Notably, the above mechanism regarding pH-dependent quinone formation remained indirect in our study, owing to the lack of direct quantitative detection of *o*-quinone intermediates.

#### 3.2.3. Effect of Metal Ions on the Conversion of 3-APA to Acrylamide in the Presence of EGCG

Trace metal ions were introduced into both the 3-APA model and the 3-APA + EGCG composite model. The mixtures were heated at 180 °C and pH 7.0 for 1, 2, 3, 4, 5, 7, and 10 min. Acrylamide formation was quantified using HPLC-UV, and the results are presented in Figure 2C.

The experimental results indicate that in the systems containing only 3-APA or with low-dose EGCG, the addition of trace metal ions did not significantly influence acrylamide formation (Figure 2(C_1_,C_2_)). Furthermore, metal ions added to the high-dose EGCG system led to a significant increase in acrylamide production compared to the control group. The enhancing effect of metal ions exhibited clear type dependence. Among them, Fe^3+^ showed the most pronounced accelerating effect, with a corresponding 71% increase in acrylamide production relative to the control, while no significant differences were observed in the Cu^2+^ and Mg^2+^ treatment groups. These findings suggest that metal ions could potentially modulate acrylamide formation via the 3-APA pathway, most likely by promoting EGCG oxidation or facilitating EGCG-metal complexation, and such catalytic behaviors are highly dependent on EGCG dosage.

Previous studies have demonstrated that polyphenol-derived *o*-quinones are generated through enzymatic and non-enzymatic oxidation pathways. Non-enzymatic autoxidation of polyphenols can be effectively accelerated by transition metal ions [34,35]. The metal-mediated autoxidation of polyphenols involves multiple sequential reactions. Catechol and pyrogallol moieties are able to coordinate with Fe^3+^ to generate *o*-quinone derivatives and Fe^2+^ [36]. The produced Fe^2+^ is further re-oxidized by oxygen to regenerate Fe^3+^, accompanied by the generation of hydroperoxyl radicals (HO_2_•). These reactive radicals abstract hydrogen from phenolic hydroxyl groups of flavan-3-ols, producing semiquinone radicals and hydrogen peroxide. Unstable semiquinone radicals readily undergo disproportionation to recover phenolic structures or further oxidized into *o*-quinone products [37]. The catechol group acts as the major reaction site for radical attack, while the pyrogallol group of EGCG exhibits comparable reactivity [36]. Based on the above literature, metal ions are speculated to indirectly enhance acrylamide accumulation by driving the oxidative transformation of EGCG, although this mechanistic link remains indirect in the present study.

From the perspective of screening conditions for acrylamide inhibition in the presence of EGCG, the experimental results offer several important insights. Firstly, specific metal ions, particularly Fe^3+^, should be avoided. Due to its strong oxidizing ability (standard redox potential E° (Fe^3+^/Fe^2+^) = +0.77 V) and high complexation affinity, Fe^3+^ exhibited the most pronounced promoting effect on acrylamide formation in systems with high EGCG concentrations. Therefore, the content of Fe^3+^ should be strictly controlled. Secondly, controlling the concentration of EGCG represents a strategy for inhibition. In environments with no or low levels of EGCG, metal ions such as Fe^3+^, Cu^2+^, and Mg^2+^ did not significantly promote acrylamide formation. This indicates that reducing EGCG concentration may help weaken metal-dependent pro-acrylamide reactions. Thirdly, the type of metal ion significantly influences acrylamide inhibition. The promoting effects of Cu^2+^ (E° = +0.34 V) and Mg^2+^ (a non-transition metal without d-orbital electrons) were considerably weaker than that of Fe^3+^. When the introduction of metal ions is unavoidable, selecting those with weaker promoting effects, such as Mg^2+^, may be more conducive to acrylamide suppression. It is particularly noteworthy that this study reveals that the metal ion-induced promotion of acrylamide formation is strictly dependent on the presence of flavan-3-ols (EGCG). This phenomenon is fundamentally distinct from the traditional catalytic role of metal ions in the Maillard reaction, suggesting that the impact of metal ions on acrylamide generation requires re-evaluation in polyphenol-rich food systems.

#### 3.2.4. Multifactor Analysis of the Conversion of 3-APA to Acrylamide Driven by EGCG

A multifactor analysis of variance (ANOVA) was conducted to evaluate the maximum acrylamide production from 3-APA conversion under various experimental conditions (n = 3). As shown in Table 1, the amount of flavan-3-ol added, the heating temperature, and the pH value were all found to be highly significant factors (*p* < 0.01). In contrast, the metal ion addition did not exhibit statistical significance, with a *p*-value of 0.332 (*p* > 0.05). The order of influence of each factor on acrylamide production was as follows: flavan-3-ol amount added > heating temperature > pH value > addition of metal ions. The non-significant outcome of metal ions in the global ANOVA may be attributed to their dose-dependent effect, which was only obvious under high-dose EGCG conditions rather than in all treatment groups.

The observed differences among treatment groups may be partially interpreted through the potential involvement of *o*-quinone species, whose formation and reactivity are highly susceptible to external environmental conditions. Firstly, flavan-3-ol dosage may serve as a critical contributor to 3-APA-related acrylamide formation. Consistent with previous findings on EC, the oxidized B-ring *o*-quinone metabolites of flavan-3-ols are capable of reacting with 3-APA via Michael addition and Schiff base formation, which potentially facilitates subsequent acrylamide generation [16]. Accordingly, excessive supplementation of EGCG is likely to increase the risk of acrylamide accumulation in the 3-APA model. Secondly, temperature may exert dual modulatory effects. High heating intensity can accelerate the oxidative transformation of EGCG into quinone-related products and may also promote the deamination of 3-APA, thereby potentially elevating the final acrylamide yield [38]. Thirdly, pH conditions can modulate the non-enzymatic oxidation progress of polyphenols. Neutral and weakly alkaline environments tend to facilitate phenolic deprotonation, enhance electron transfer efficiency, and accelerate radical-mediated oxidation reactions [31]. Such pH-dependent oxidation processes may favor *o*-quinone accumulation and further boost acrylamide production. Fourth, the pro-oxidant effect of metal ions is highly conditional and substrate-dependent. Although metal ions such as Fe^3+^ and Cu^2+^ did not affect acrylamide levels across all groups, they may potentially accelerate the initial oxidation of polyphenols and enhance quinone formation only when sufficient EGCG substrate is provided [39,40,41]. This conditional trend may reasonably account for the inconsistent performance of metal ions between overall ANOVA and high-dose EGCG treatments.

In this study, different temperatures, pH values, and metal ion types were set to simulate various food processing conditions. The differences in acrylamide production from 3-APA deamination in the presence of EGCG were compared and analyzed. Analysis of variance clarified the contribution weights of each influencing factor as follows: flavan-3-ol addition amount > heating temperature > pH value > metal ion addition. To prevent EGCG from promoting acrylamide formation via the 3-APA path-way, it is necessary to avoid high-temperature and prolonged processing, maintain a slightly acidic environment to suppress phenolic oxidation and the nucleophilicity of 3-APA, and incorporate metal chelators to inhibit Fe^3+^ catalysis.

### 3.3. Mechanism for the Condition-Dependent Conversion of 3-APA to Acrylamide in the Presence of EGCG

#### 3.3.1. Identification of EGCG Quinone in the Reaction System

The influence of temperature, pH, and metal ions on the deamination of 3-APA to form acrylamide in the presence of EGCG indicated that environmental conditions might modulate acrylamide formation by affecting the generation efficiency of *o*-quinones and their subsequent reactivity. As a potential key intermediate, *o*-quinone may contribute to the reaction between EGCG and 3-APA that facilitates acrylamide formation. Therefore, verifying its existence helps to partially explain the underlying reaction mechanism. However, *o*-quinones are highly unstable and reactive [42]. To improve the reliability of qualitative analysis, o-phenylenediamine (OPD) was selected as a nucleophilic derivatization reagent in this study. It specifically reacted with *o*-quinone intermediates to form stable phenazine derivatives suitable for qualitative detection [43].

**Figure 3:**
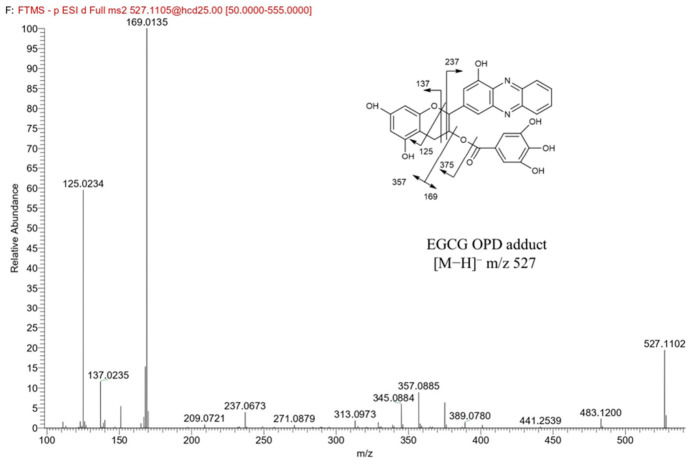
MS/MS fragmentation spectrum of EGCG quinone-OPD adduct ([M–H]^−^ *m*/*z* 527) in negative ionization mode and the mass spectral fragmentation pathways.

UPLC-MS/MS analysis detected the EGCG-OPD derivative ([M–H]^−^ *m*/*z* 527.1105) at a retention time of 24.15 min in the reaction system following derivatization with o-phenylenediamine (Figure 3). The major fragment ions included [M–H]^−^ *m*/*z* 125, 137, 169, 237, 357, and 375. Among these, the cleavage of bonds 1 and 3 in the C ring generated fragments 1,3A^−^ with *m*/*z* = 137 and 1,3B^−^ with *m*/*z* = 237, respectively. Ions with *m*/*z* 169 and 357 corresponded to the loss of gallic acid and the remaining part, while *m*/*z* 375 resulted from a loss of galloyl group. The ion with *m*/*z* 125 represented 1,4A^−^ after the cleavage of C ring at bonds 1 and 4. The identification of the EGCG-OPD structure was supported by accurate mass measurements, with mass errors ranging from 1.2 to 8.1 ppm (0.3–1.0 mDa). These results verified the formation of EGCG-derived quinone intermediates in the reaction system containing 3-APA and EGCG.

#### 3.3.2. Correlation Analysis of the Redox State of EGCG and the Formation of Acrylamide

Owing to its highly conjugated and unstable nature, the quinone form of EGCG is difficult to quantify directly. However, its dynamic oxidation can be precisely characterized by electrochemical methods, which provide high sensitivity and permit real-time monitoring [44,45,46]. This study first established a calibration between the oxidation peak current intensity of phenolic hydroxyl groups and EGCG concentration. The redox state of EGCG in the 3-APA + EGCG system was then examined under varying conditions. Ultimately, a correlation between the EGCG consumption rate and the acrylamide formation rate was determined, providing valuable clues to partially clarify the potential mechanism through which EGCG influences 3-APA deamination.

**Figure 4:**
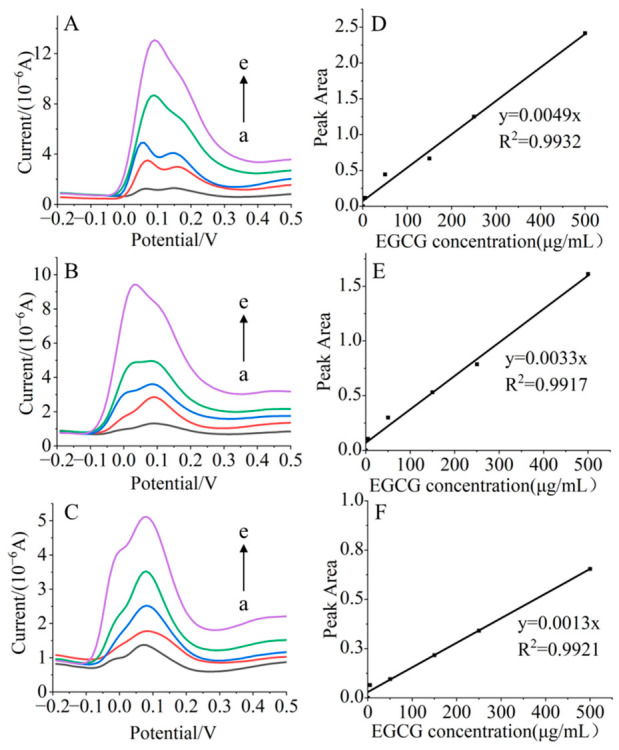
Differential pulse voltammetry (DPV) profiles and calibration curves of EGCG at different pH values. Panels (**A**–**C**) show the DPV diagrams obtained at pH 6, 7, and 8, respectively. Panels (**D**–**F**) present the corresponding linear calibration plots of peak area versus EGCG concentration at each pH. In all diagrams, curves a to e correspond to EGCG concentrations of 5, 50, 150, 250, and 500 µg/mL, respectively.

As shown in Figure 4, the characteristic oxidation peaks of EGCG were observed within the potential range of 0–0.3 V. As the pH of the solution increased, the anodic peak potential slightly shifted toward a negative value. According to the literature, the appearance of this shoulder peak was due to the oxidation of the 3′,4′-dihydroxy group on the B ring of EGCG, corresponding to the formation of the semiquinone radical and the quinone, respectively [47]. The oxidation current increased with the increase in EGCG concentration (Figure 4A–C, a → e). Linear relationships of peak areas on concentration of EGCG were observed at pH 6, 7, and 8 (Figure 4D–F), thus allowing for the accurate quantification of the residual EGCG in the system during the reaction under different conditions.

**Figure 5:**
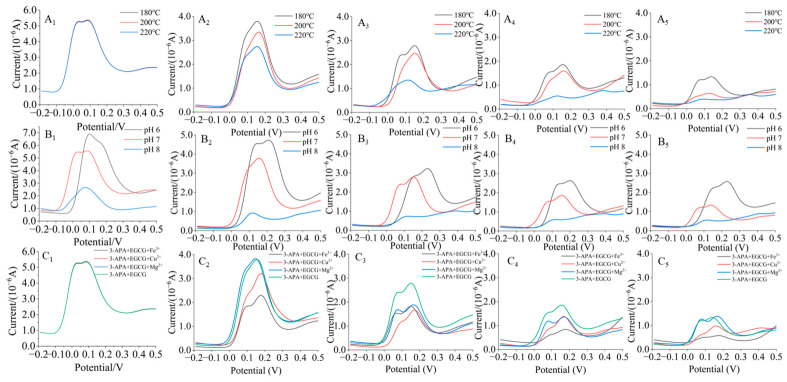
DPV diagrams of the 3-APA + EGCG system under different reaction conditions. Panels (**A_1_**–**C_5_**) show the effect of temperature, pH, and metal ions, respectively. The subscripts 1, 2, 3, 4, and 5 denote the reaction time for 0, 1, 3, 5, and 10 min, respectively.

Figure 5 presents the DPV analysis results for samples reacted under different conditions. Consistently, lower residual amounts of EGCG were observed at elevated temperatures, higher pH, or in the presence of metal ions. Kinetic fitting analysis of the initial 0–5 min period showed that acrylamide formation followed zero-order kinetics, while EGCG consumption followed first-order kinetics (Table 2). Notably, at pH 8, EGCG consumption deviated from first-order kinetics, which may be attributed to the accelerated self-polymerization of EGCG-derived *o*-quinones under alkaline conditions. The calculated EGCG consumption rate constant (*k*_EGCG_) increased with rising temperature or pH, indicating a faster oxidation of EGCG to *o*-quinones. Similarly, the addition of metal ions, compared to the ion-free control, led to an increased *k*_EGCG_, suggesting a catalytic role of metal ions in promoting EGCG oxidation and conversion.

**Figure 6:**
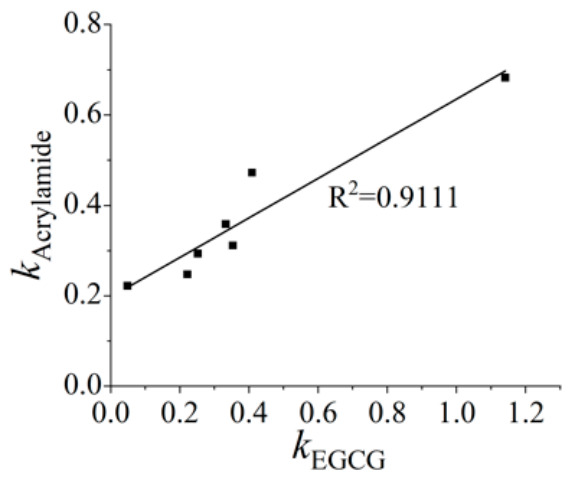
Correlation analysis of *k*_EGCG_ and *k*_acrylamide_ in the 3-APA + EGCG system.

The correlation between acrylamide formation rate and EGCG consumption rate was further analyzed (Figure 6). A statistically significant positive correlation was observed (*p* < 0.05), confirming that acrylamide formation is directly linked to EGCG consumption in the 3-APA + EGCG model system. As the oxidation peak in the DPV curve corresponded to phenolic hydroxyl groups, a decrease in peak area signified the formation of quinones. This result demonstrated that reaction conditions ultimately influenced acrylamide yield by modulating the redox state of EGCG. Specifically, temperature, pH, and metal ions modified the oxidation efficiency of EGCG, which in turn dictated its capacity to promote the conversion of 3-APA to acrylamide.

### 3.4. Mechanism for the Condition-Dependent Inhibition of EGCG on Acrylamide Formation in the Maillard Reaction

Previous studies have established EGCG as the most potent acrylamide inhibitor among flavan-3-ols [10]. To advance its practical application, this study focused on optimizing the relevant reaction conditions. The findings from Section 3.1 revealed that EGCG achieved the maximum inhibition of acrylamide formation in the Maillard reaction under a combination of moderate temperature, acidic pH, and the absence of metal ions. This is consistent with previous reports that acidic conditions stabilize EGCG by suppressing autoxidation, while metal ions (Fe^3+^, Cu^2+^) markedly accelerate its degradation [48,49]. Meanwhile, the data in Section 3.2 confirmed that the potential pro-acrylamide effect of EGCG via the 3-APA pathway was also largely weakened under the same mild conditions. Such consistent trends suggest that environmental factors collectively modulate EGCG’s inhibitory efficacy, at least partially by regulating its oxidative behavior within the 3-APA reaction pathway.

**Figure 7:**
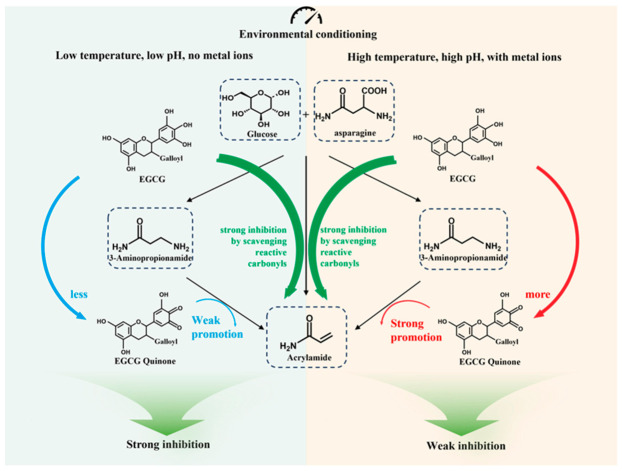
Mechanism for the condition-dependent inhibition of EGCG on acrylamide formation in the Maillard reaction.

Mechanistically, EGCG exhibits a robust carbonyl-scavenging capacity, which is the primary driver for acrylamide mitigation in Maillard systems. Under favorable conditions (low temperature, weakly acidic pH and absence of catalytic metal ions), the oxidative transformation of EGCG and its subsequent promotion of 3-APA deamination are effectively suppressed. In such scenarios, the adverse pro-acrylamide side effect is minimized, thereby maximizing the net inhibitory efficiency against Maillard-derived acrylamide (Figure 7).

As a natural bioactive constituent, EGCG is inherently thermolabile. Significant degradation begins above 80 °C, and rapid oxidation occurs beyond 120 °C, potentially leading to a loss of active EGCG during intensive thermal procedures like baking or frying [50,51]. Consequently, food processing should incorporate strategic addition methods to mitigate thermal damage. For instance, introducing high-purity or microencapsulated EGCG at lower temperature stages is an effective approach to preserve its structural integrity and bioactivity [52,53]. Furthermore, the matrix environment is critical. While low pH inhibits autoxidation, neutral to alkaline environments coupled with transition metal ions significantly facilitate EGCG decomposition [48,49].

Based on these mechanistic insights, targeted strategies can be implemented to optimize EGCG efficiency in food production. First, emerging non-thermal technologies (e.g., high-pressure processing) could partially replace conventional thermal operations to prevent high-temperature-induced phenolic oxidation. Second, maintaining a weakly acidic or near-neutral pH during processing is vital for preserving EGCG activity. Third, the addition of food-grade chelators (e.g., phytic acid or EDTA) can sequester Fe^3+^ ions and thereby block their catalytic role in EGCG autoxidation. Overall, the integrated optimization of processing temperature, pH, and the food matrix is essential for the effective and stable application of EGCG as an acrylamide mitigant in complex food systems.

## 4. Conclusions

Environmental conditions modulated the redox state of EGCG, which further affected the degradation of 3-APA and thereby partially mediated the inhibitory efficiency of EGCG against acrylamide formation. Lower temperature, acidic pH, and metal-ion-free environments improved EGCG’s inhibitory capacity. ANOVA results revealed a clear order in the relative contributions of influencing factors: EGCG concentration exerted the primary impact, followed by temperature, pH, and metal ions. Electrochemical analysis indicated a positive correlation between acrylamide generation from 3-APA and EGCG’s consumption of phenolic hydroxyl groups, suggesting that reaction conditions regulate the *o*-quinone pathway. Mass spectrometry confirmed the EGCG *o*-quinone-OPD adduct, supporting this pathway. EGCG exhibited optimal inhibition (91%) at 180 °C and pH 6 without metal ions. Thus, low temperature, non-alkaline conditions, and exclusion of metal contamination are recommended for improving EGCG’s anti-acrylamide performance in food processing. However, these research results are based on simplified model systems and are still far from the actual food system. Further verification in a real food environment is necessary.

## Figures and Tables

**Table 1:** Analysis of variance for the amount of acrylamide produced in the reaction between 3-APA and EGCG under different conditions.

Source of Variance	Sum of Squares	Degrees of Freedom	Mean Square	F-Value	*p*-Value	Effect Size (η_p_^2^)	Significance
Model	55.986	10	5.599	46.052	<0.0001	0.924	**
Addition Amount	22.937	2	11.468	94.333	<0.0001	0.832	**
Heating Temperature	2.252	2	1.126	9.261	0.001	0.328	**
pH Value	1.640	2	0.820	6.746	0.003	0.262	**
Metal Ion Addition	0.428	3	0.143	1.175	0.332	0.085	
Error	4.620	38	0.122				
Total	60.606	48					

Notes: “*” indicates a significant difference at the 0.05 level (*p* < 0.05); “**” indicates an extremely significant difference at the 0.01 level (*p* < 0.01); no mark indicates no significant difference (*p* > 0.05).

**Table 2:** Fitting curve parameters of EGCG consumption (*k*_EGCG_) and acrylamide generation (*k*_acrylamide_) under different reaction conditions.

Reaction Condition		AA Production			EGCG Consumption		
		Regression Equation	*k*_AA_	R^2^	Regression Equation	*k*_EGCG_	R^2^
Temperature	180 °C	y = 0.2215x − 0.2481	0.2215	0.9928	lny = −0.2479x + 5.4325	0.2479	0.9751
	200 °C	y = 0.2516x − 0.3166	0.2516	0.9608	lny = −0.2936x + 5.3811	0.2936	0.9529
	220 °C	y = 1.1412x − 1.1416	1.1412	0.9314	lny = −0.6830x + 5.4852	0.6830	0.9952
pH Value	pH 6	y = 0.0481x − 0.0798	0.0481	0.9795	lny = −0.2227x + 5.3808	0.2227	0.9045
	pH 7	y = 0.2215x − 0.2481	0.2215	0.9928	lny = −0.2479x + 5.4325	0.2479	0.9751
	pH 8	y = 0.2969x − 0.4160	0.2969	0.9751	lny = −0.6878x + 4.5565	0.6878	0.7462
Ion Type	Control	y = 0.2215x − 0.2481	0.2215	0.9928	lny = −0.2479x + 5.4325	0.2479	0.9751
	Fe^3+^	y = 0.4084x − 0.4947	0.4084	0.9938	lny = −0.4726x + 5.3503	0.4726	0.9226
	Cu^2+^	y = 0.3323x − 0.3853	0.3323	0.9805	lny = −0.3590x + 5.3482	0.3590	0.9487
	Mg^2+^	y = 0.3529x − 0.4809	0.3529	0.9832	lny = −0.3118x + 5.4258	0.3118	0.9637

## Data Availability

The original contributions presented in this study are included in the article. Further inquiries can be directed to the corresponding author.

## Abbreviations

The following abbreviations are used in this manuscript:

AA	Acrylamide
EGCG	Epigallocatechin gallate
EC	Epicatechin
3-APA	3-Aminopropionamide
DPV	Differential pulse voltammetry
